# Motivations for sexual risk behaviors among older men in Shanghai, China: a qualitative study

**DOI:** 10.1186/1471-2458-14-802

**Published:** 2014-08-07

**Authors:** Yanqiu Zhou, Yingying Ding, Kaikan Gu, Xiaonian Lu, Meiyang Gao, Na He

**Affiliations:** Jingan District Center for Disease Control and Prevention, Shanghai, China; Department of Epidemiology, School of Public Health, Fudan University, and The Key Laboratory of Public Health Safety of Ministry of Education, Shanghai, 200032 China; Department of Dermatology, Huashan Hospital, Fudan University, Shanghai, China

**Keywords:** HIV/AIDS, Motivation, Sexual risk behaviors, Older men, China

## Abstract

**Background:**

China’s population is quickly aging and this trend is expected to continue. Thus it is important to develop HIV interventions to help protect older Chinese from infection. Limited information exists regarding sexual risk behaviors and associated personal motivations among persons aged 50 and over in China.

**Methods:**

In-depth interviews were conducted with 12 HIV-infected and 14 uninfected men aged 50 and over in Shanghai, China.

**Results:**

More than 71% of heterosexual participants had engaged in commercial sex, 37.5% either had engaged in casual sex or had a steady extramarital partner. All gay/bisexual participants had engaged in casual sex with men, and 16.7% had engaged in commercial sex. Personal motivations associated with sexual risk behaviors included sexual desire and interest in sex remaining high at an older age, unfulfilled sexual desires within marriage, homosexual or bisexual orientation, need to socialize with others, peer influence, personal choice of “hobby”, and financial freedom.

**Conclusions:**

This study sheds light on the sexual needs of older people. Our findings underscore the need for both greater education in order to reshape societal perceptions of sexuality among older adults and prevention strategies to help the older male population maintain a healthy sexual life.

## Background

Despite the popular belief that older adults do not constitute a risk population for HIV infection, this population is most certainly at risk [1]. Recent estimates have shown that new HIV cases in China among the 50+ age group increased significantly from 2000 to 2011. In the 50–64 age group the number of cases increased from 1.6% of the total number of reported cases in 2000 to 13.8% in 2011, while in the 65+ age group the number of cases increased from 0.34% in 2000 to 7.3% in 2011 [2]. As China’s population continues to age quickly, it is important to develop HIV interventions that will help protect older Chinese populations from infection. Unfortunately, limited information exists regarding sexual risk behaviors and associated motivations among persons aged 50 years and over, particularly in China.

Many studies have shown that older adults remain sexually active and maintain a level of sexual desire, even though interest in sex and frequency of intercourse generally decline with age [3–6]. A survey of 1,550 women and 1,455 men in the United States found that 73% of those aged 57–64 were sexually active, as were 53% of those aged 65–74 and 26% of those aged 75–85 [4]. Older men were more likely to be sexually active than older women [4]. Moreover, there is increasing evidence that older people, like their younger counterparts, engage in risk-taking sexual behaviors [7–9]. In a study of older men aged 49–80, one quarter had more than one sexual partner in the prior six months. Older individuals also are less likely than their younger counterparts to use condoms during sex [10, 11].

This article reports findings from in-depth, qualitative interviews conducted with a total of 26 HIV-infected and HIV-uninfected men aged 50 years and over in China. The present analyses sought to explore the range of personal motivators associated with sexual risk behaviors among older male adults. Greater knowledge regarding patterns of and motivations for sexual risk behaviors will aid in promoting better care and prevention as well as development of age-specific interventions for older adults.

## Methods

### Participants and recruitment

In 2010, we interviewed 26 older men living in Shanghai – 12 HIV-infected and 14 HIV-uninfected – in order to explore causes and patterns of sexual risk behaviors among this population. The 14 uninfected participants were recruited from a voluntary counseling and testing (VCT) clinic. Seven infected participants were invited by health staff to participate, while five infected participants were recruited by HIV-infected volunteers. Participants were eligible if they were male, were aged 50 years or older. Each was paid $16 USD for completing the interview. All participants were given written consent. Beforehand the study protocol was reviewed and approved by the Institutional Review Board at Fudan University. This study adheres to the RATS guidelines on qualitative research [12].

### Interview methods

Each semi-structured interview covered four topics: (1) background information (e.g., age, marital status, education, occupation, sexual orientation, and HIV status), (2) sexual risk behaviors, mainly focusing on extramarital sex (e.g., engaging in casual sex or commercial sex, and having steady extramarital partners), (3) condom use with sexual partners, and (4) motivations associated with sexual risk behaviors. For HIV-infected participants, all questions from topics (2) to (4) pertained to sexual risk behaviors, condom use, and associated motivators *before* confirmation of HIV diagnosis. All interviews were audio-recorded with the permission of respondents, and then transcribed verbatim. The resulting transcripts were examined by the author through an iterative process in order to identify recurrent themes and develop corresponding codes to be applied to the text. Themes were identified both inductively and deductively based on issues extrapolated from the data.

## Results

### Participant profiles

Table 1 shows sample characteristics of the 26 older men who participated. All participants were between the ages of 50 and 75 years old, and most had at least a high school education (65.4%) and were currently employed (61.5%). The majority were married (61.5%), while 15.4% were never married and 23.1% were divorced or widowed. Two-thirds (66.7%) of HIV-infected and 35.7% of HIV-uninfected participants self-identified as gay or bisexual. All HIV-infected participants had been diagnosed at age 50 or older (range: 50 to 71).

**Table 1:** **Sample description (** ***N*** **= 26)**

Characteristic	HIV-infected(***n*** = 12)		HIV-uninfected(***n*** = 14)	
	n	%	n	%
Age				
50-59	6	50.0	12	85.7
60-69	4	33.3	2	14.3
70-75	2	16.7	0	0.0
Education				
Less than high school	6	50.0	3	21.4
High school or equal	3	25.0	4	28.6
College or above	3	25.0	7	50.0
Marital status				
Never married	3	25.0	1	7.1
Married	8	66.7	8	57.1
Divorced or widowed	1	8.3	5	35.7
Employment status				
Employed	5	41.7	11	78.6
Retired, unemployed	7	58.3	3	21.4
Sexual orientation				
Heterosexual	8	66.7	4	28.6
Gay or bisexual	4	33.3	10	71.4
Age at HIV diagnosis				
50-59	6	50.0	-	-
60-69	4	33.3	-	-
70-75	2	16.7	-	-

### Sexual risk behaviors

Among heterosexual participants, 100% reported engaging in sexual risk behaviors, including commercial sex (n = 10; 71.4%), and casual sex or having a steady extramarital partner (n = 6; 37.5%). More than half of heterosexual participants (n = 8; 57.1%) reported never using a condom when they had sex, while four (28.6%) reported using a condom sometimes and two (14.3%) reported using a condom most of the time.

Among gay/bisexual participants, 100% reported engaging in casual sex with men, and one (8.3%) engaged in casual sex with women. Only two (16.7%) reported engaging in commercial sex: one engaged in selling sex to men, the other engaged in buying sex from a prostitute. One (8.3%) never married and one (8.3%) divorced participant reported having a stable male sexual partner. Regarding condom use, only one (8.3%) reported using a condom when having sex; the other 11 (91.7%) reported never using a condom.

Among gay/bisexual participants, the most common locations for finding sex partners were bathhouses, bars, parks, and the Internet. Among heterosexual participants, the most common locations were bathhouses, brothels, parks, and the street. Among gay/bisexual participants, sexual encounters occurred most frequently in hotels, bathhouses, their own or their partner’s house, and parks. Among heterosexual participants, sex tended to take place in bathhouses, brothels, hotels, and their partner’s house.

### Motivators associated with sexual risk behaviors

Participants reported engaging in sexual risk behaviors due to: (1) sexual desire or interest in sex remaining high at an older age, (2) unfulfilled sexual desires within marriage, (3) homosexual or bisexual orientation, (4) need to socialize with others, and (5) peer influence. A small subset engaged in sexual risk behaviors due to: (6) personal choice of “hobby” and (7) financial freedom.

#### Sexual desire/interest remaining high at an older age

Participants commonly reported maintaining an interest in sexual activity and felt a high level of sexual desire at an older age. For example:

*I’m annoyed that I have an interest in sex even though I’m almost 60 years old and am going to be retired. (N10, 58 ys, HIV-, heterosexual)*

*I go out to visit a “xiaojie” (prostitute) because I have especially strong sexual desires. (N5, 60 ys, HIV-, heterosexual)*

#### Unfulfilled sexual desires within marriage

Almost all of the heterosexual participants (whether HIV-infected or -uninfected) stated that the primary motivator for seeking extramarital sex was that their spouses could not fulfill their sexual desires. Many participants reported that their spouses had no interest in sexual activity, especially after getting older. For instance:

*My wife and I are sexually incompatible. I want sex frequently but she wants sex less frequently. (N5, 60 ys, HIV-, heterosexual)*

*My wife did not allow me to touch her after menopause. I still had sexual needs; at first I [masturbated]. The first time [having sex] was by chance: I was having a shower in a bathhouse when a “xiaojie” (prostitute) asked me if I wanted a massage. I couldn’t resist and then we had sex. (N10, 58 ys, HIV-, heterosexual)*

*I had [sex with a prostitute] mainly because my wife didn’t want to have sex. She said “if you want sex, you can go out and find it”, so I went out… (N11, 56 ys, HIV-, heterosexual)*

In some cases, participants reported their spouses could not have sex due to illness:

*My wife got cervical cancer and many other diseases and thus she could not have sex. Then one day I met a colleague … by accident [and] he and I had sex. (G1, 68 ys, HIV+, homosexual)*

*My wife had a cerebral infarction, but I still had sexual desires and I cannot resist the temptation [to visit prostitutes]. (G12, 75 ys, HIV+, heterosexual)*

One participant reported that the reason for seeking extramarital sex was that he had divorced.

*I began to engage in extramarital sex after I got divorced. (N6, HIV-, 53 ys, heterosexual)*

#### Homosexual or bisexual orientation

Among gay/bisexual participants, the main reason for engaging in extramarital sex or having multiple sexual partners was due to their homosexual or bisexual orientation. Eleven of the 14 gay/bisexual participants entered into marriage due to social and familial pressures when they were young. But as they aged, social pressures at work and in life reduced while simultaneously society was becoming increasingly tolerant of homosexuality. As a result, they began to spend more time with other homosexuals and engaged in gay activities in pursuit of their own pleasures.

*I was homosexually-oriented when I was little. Then, at a time when conditions and environmental factors favored it, I began to engage in homosexual behaviors. (N7, 55 ys, HIV-, bisexual)*

*Accidentally I met a gay man while playing Mahjong with friends [and] after several years, he asked “How come you and your wife don’t have sex?”, then I had sex with him. (G11, 66 ys, HIV+, homosexual)*

*Sometimes I’m disgusted with homosexuals, but sometimes I like it; otherwise I would not be as I am now (married and bisexual); I have a good relationship with my wife. (N9, 59 ys, HIV-, bisexual)*

Two of three never-married gay participants reported engaging in casual sex mainly because they had no stable sexual partner(s).

#### Peer influence

A few participants reported being motivated to engage in sexual risk behaviors because of peer influence. They went out with others to visit prostitutes. For example:

*This is a human physiological need; we have sexual needs and desires. If you become part of a group where everyone is [buying sex] and doing so together, it becomes normal, and so you do it together with them. (G12, 75ys, HIV+, heterosexual)*

*We friends hang out together, and then all of us visit a “xiaojie” (prostitute). (G3, 61 ys, HIV+, heterosexual)*

*It is not a joke that my biologic brother and I drove together to visit a “xiaojie” (prostitute). (G9, HIV+, 57 ys, bisexual)*

#### Need to socialize with others

Several participants reported being motivated to engage in sexual risk behaviors in order to socialize with others. Some were not yet retired and needed to socialize for business and work reasons, which promoted engaging in risky sexual behaviors.

*Sometimes [business partners] offer prostitutes to entertain you. They pay and the prostitutes don’t leave, though mainly [the reason I do this is] because I cannot stop [visiting prostitutes]. (G2, 59 ys, HIV+, heterosexual)*

*I’ve [visited prostitutes] since I started in business. If I didn’t work in business, I wouldn’t do that kind of thing. I think the primary reason is the influence of the social environment. Everyone [visits prostitutes], so I follow along. (G3, 61ys, HIV+, heterosexual)*

*One time I went to (a particular city) on business, and my [business partner] said “let’s change the vibe”, so we went to a club where there were sexy dance performances by men. (G9, 57ys, HIV+, bisexual)*

#### Personal choice of “hobby”

For some participants, engaging in extramarital sex was akin to a personal hobby since they were younger. Several heterosexual participants identified visiting a prostitute as a personal hobby, For example:

*Women are my lifelong hobby; I spend all my money on women. Only on that day when I lie on the bed and cannot walk will [having sex] be over. (G10, 71 ys, HIV+, heterosexual)*

*Some people like growing flowers but I like [visiting prostitutes], so I know [myself]. (G12, 75ys, HIV+, heterosexual)*

One bisexual participant identified having homosexual partners as a personal hobby.

*My thinking is simple: in sexual relationships, your spouse is primary. The homosexual relationship is the side relationship, like having another hobby. (N7, 55 ys, HIV-,bisexual)*

#### Financial freedom

Financial freedom was also a reason for engaging in extramarital sex among older men. Among these men aged 50 or older who were neither poor nor rich, some enjoyed spending money to satisfy themselves. For example:

*I don’t drink, I only smoke sometimes. I don’t go to clubs and restaurants, have no need to socialize, and am not in the habit of saving money. Think what I could spend money on: that is [visiting prostitutes]. (G10, 71 ys, HIV+, heterosexual)*

## Discussion

The purpose of this study was to explore the personal motivators associated with engaging in sexual risk behaviors among older men in China. Respondents in our study reported engaging in a variety of HIV risk-related sexual activities, including low rates of condom use and having casual sex partners, commercial sex partners or stable extramarital sex partners, which corroborate findings obtained previously among older adults [7, 8, 13]. Given both the growing number of new HIV cases reported among those aged 50 years or older in China [1] and that all prevention interventions target young adults while neglecting older adults, there is an urgent need for HIV prevention interventions designed specifically for older adults.

We noted that heterosexual older men were more likely to engage in commercial sex, while gay/bisexual older men were more likely to engage in casual sex; this pattern is similar to that of heterosexual and gay/bisexual young men in China [14, 15]. Such a pattern may depend primarily on the availability of casual and commercial sex partners; it is easier for gay/bisexual men to find casual sex partners but easier for heterosexual older men to find commercial sex partners. This difference underscores the need to tailor HIV prevention interventions specifically for heterosexual and gay/bisexual older persons.

The most commonly-reported personal motivators for engaging in extramarital sex among heterosexual participants were continued high levels of sexual desire and interest into later life and unfulfilled sexual desires within marriage. This finding was consistent with those from other studies where a majority of older men remained sexually active and considered sex to be an important part of life [4]. China has seen increasingly open attitudes toward sex and greater freedom of sexual expression in recent years, yet there remains a stereotype that older people have no sexual interest and desire, and a belief that older adults should not engage in any sexual behaviors. Such misconceptions might play a role in preventing older persons from enjoying healthy and safe sex lives. Compared with older men, older women tend to have less sexual desire and interest [4]. Our participants reported this to be the major reason for sexual incompatibility within their marriage, and further motivated men to engage in extramarital sex. Therefore, sexuality among older adults should receive greater societal attention. Efforts to reshape perceptions of sexuality and aging among both older people and society in general are needed in all forms of media. Greater education of health care providers – particularly those working with older people – will increase understanding and awareness of older peoples’ sexual needs.

The primary motivator for gay/bisexual participants to engage in sexual risk behaviors was their homosexual/bisexual orientation. Unlike in Western societies where a small percentage (an estimated 1.5–6%) of men who have sex men (MSM) are married to women [16, 17], approximately 70-90% of MSM in China marry a woman due to societal and parental pressures [18, 19]. We found that 78.5% of our older gay/bisexual participants were ever married, which is higher than rates found for younger MSM in China [19, 20]. Similar findings had been observed previously in China, where older MSM were more likely to have married [19]. With greater tolerance for homosexuality, younger MSM likely feel less societal and familial pressure to marry, while a growing number of older, married MSM are beginning their own sexual relationships with other males.

Similar to the motivations reported for initiating drug use among drug users [21, 22], peer influence and the need to socialize with others were two common motivators cited by older persons for engaging in sexual risk behaviors. Other motivators include personal choice of “hobby” and financial freedom. Older men in large and medium-sized cities in China usually experience less financial pressure compared to younger men in paying for housing and raising children. Thus, it is important for older adults – particularly those who are retired – to develop healthy hobbies and interests. Family and community members should take a more active role in helping older adults to live healthy and happy lives. The community, for example, could organize more social activities for older adults.

There are limitations to our data which should be considered when interpreting and evaluating the results. Because these data were gathered from a convenience sample, the findings should not be considered generalizable. All participants were either HIV-infected or recruited from VCT centers, thus all had engaged in sexual risk behaviors. However, our findings could reflect the motivations for engaging in sexual risk behaviors among the older male population. Also, our results were based on self-reported data, which depend on the veracity of the respondents.

## Conclusions

Our study’s findings contribute to the vital mission of understanding the sexual risk behaviors and associated personal motivations among older males in China. The data suggest that older people are sexually active and engage in sexual risk activities. Education is needed in order to reshape societal perceptions of sexuality among older adults. Although sexual activity among these older adults is now more widely accepted, it still should be remembered that sexual risk encounters may result in HIV/STD infection. Our findings also emphasize the need for developing prevention and intervention programs that will help the older male population maintain healthy sexual lives.

## References

[1] Goodroad BK (2003). HIV and AIDS in people older than 50: a continuing concern. J Gerontol Nurs.

[2] China Ministry of Health (2012). 2012 China AIDS Response Progress Report.

[3] Kazer MW (2013). Sexuality in older adults: changing misconceptions. J Gerontol Nurs.

[4] Lindau ST, Schumm LP, Laumann EO, Levinson W, O’Muircheartaigh CA, Waite LJ (2007). A study of sexuality and health among older adults in the United States. NEJM.

[5] Reece M, Herbenick D, Schick V, Sanders SA, Dodge B, Fortenberry JD (2007). Condom use rates in a national probability sample of males and females ages 14 to 94 in the United States. J Sex Med.

[6] Wang TF, Lu CH, Chen IJ, Yu S (2008). Sexual knowledge, attitudes and activity of older people in Taipei, Taiwan. J Clin Nurs.

[7] Cooperman NA, Arnsten JH, Klein RS (2007). Current sexual activity and risky sexual behavior in older men with or at risk for HIV infection. AIDS Educ Prev.

[8] Foster V, Clark PC, Holstad MM, Burgess E (2012). Factors associated with risky sexual behaviors in older adults. J Assoc Nurses AIDS Care.

[9] Illa L, Brickman A, Saint-Jean G, Echenique M, Metsch L, Eisdorfer C, Bustamante-Avellaneda V, Sanchez-Martinez M (2008). Sexual risk behaviors in late middle age and older HIV seropositive adults. AIDS Behav.

[10] Reece M, Herbenick D, Schick V, Sanders SA, Dodge B, Fortenberry JD (2010). Sexual behaviors, relationships, and perceived health among adult men in the United States: results from a national probability sample. J Sex Med.

[11] Schick V, Herbenick D, Reece M, Sanders SA, Dodge B, Middlestadt SE, Fortenberry JD (2010). Sexual behaviors, condom use, and sexual health of Americans over 50: implications for sexual health promotion for older adults. J Sex Med.

[12] *Qualitative Research Review Guidelines – RATS*. http://www.biomedcentral.com/authors/rats

[13] Xu Y, Hei F, Hui S, Chen H, Liang L, Zhu Q, Qin Q, Ding Z, Li P, Wang L (2011). Comparison of high-risk behaviors among middle-aged and elderly HIV/AIDS patients before and after diagnosis and the impact factors. Chin J AIDS STD.

[14] Liu H, Yang H, Li X, Wang N, Liu H, Wang B, Stanton B (2006). Men who have sex with men and human immunodeficiency virus/sexually transmitted disease control in China. Sex Transm Dis.

[15] Zhao R, Gao H, Shi X, Tucker JD, Yang Z, Min X, Qian H, Duan Q, Wang N (2005). Sexually transmitted disease/HIV and heterosexual risk among miners in townships of Yunnan Province, China. AIDS Patient Care STDS.

[16] Xu F, Sternberg MR, Markowitz LE (2010). Men who have sex with men in the United States: demographic and behavioral characteristics and prevalence of HIV and HSV-2 infection: results from national health and nutrition examination survey 2001–2006. Sex Transm Dis.

[17] Weinhardt LS, Kelly JA, Brondino MJ, Rotheram-Borus MJ, Kirshenbaum SB, Chesney MA, Remien RH, Morin SF, Lightfoot M, Ehrhardt AA, Johnson MO, Catz SL, Pinkerton SD, Benotsch EG, Hong D, Gore-Felton C, National Institute of Mental Health Healthy Living Project Team (2004). HIV transmission risk behavior among men and women living with HIV in 4 cities in the United States. JAIDS.

[18] Zhang B, Li X, Hu T, Liu D, Shi T (2000). HIV/AIDS interventions targeting men who have sex with men (MSM): theory and practice. Chin J STD/AIDS Prev Control.

[19] Wang C, Yang Y, Lu G, Yang X, Ge L, Zhao H (2007). Intervent male male sexual contact AIDS high dangerous behavior and evaluate effection of intervention. Chin J Health Lab Tech.

[20] Choi K, Liu H, Guo Y, Han L, Mandel JS, Rutherford GW (2003). Emerging HIV-1 epidemic in China in men who have sex with men. Lancet.

[21] Ding Y, He N, Detels R (2013). Circumstances of initiation into new-type drug use among adults in Shanghai: are there differences by types of first new-type drug used?. Drug Alcohol Depend.

[22] Tucker JS, de la Haye K, Kennedy DP, Green HDJ, Pollard MS (2014). Peer influence on marijuana use in different types of friendships. J Adolesc Health.

[CR23] The pre-publication history for this paper can be accessed here:http://www.biomedcentral.com/1471-2458/14/802/prepub

